# Measuring Tumour Imatinib Concentrations in Gastrointestinal Stromal Tumours: Relevant or Redundant?

**DOI:** 10.3390/cancers15112875

**Published:** 2023-05-23

**Authors:** Eline L. Giraud, Loek A. W. de Jong, Erik van den Hombergh, Suzanne E. J. Kaal, Nielka P. van Erp, Ingrid M. E. Desar

**Affiliations:** 1 Radboud University Medical Centre, Department of Pharmacy, 6500 HB Nijmegen, The Netherlands; 2 Radboud University Medical Centre, Department of Medical Oncology, 6500 HB Nijmegen, The Netherlands

**Keywords:** imatinib, neoadjuvant treatment, gastrointestinal stromal tumours, GIST, tyrosine kinase inhibitors, tumour drug concentrations, tumoral drug concentrations, pathological treatment response

## Abstract

**Simple Summary:**

It is largely unknown how imatinib distributes within gastrointestinal stromal tumours (GISTs) and whether imatinib plasma concentrations correlate with tumour concentrations, whilst plasma concentrations are used to optimize treatment. In this exploratory study imatinib tumour concentrations were measured in different tumour regions after neoadjuvant treatment. The goal of this study was to reveal tumour distribution patterns and to investigate the possible correlation between plasma and tumour concentrations. Imatinib appears to accumulate in tumour tissue since tumour concentrations were higher compared to plasma concentrations. No clear distribution pattern within the tumour could be identified. Interpatient variability in tumour concentration was almost threefold higher than interindividual variability in plasma concentration. No correlation between tumour and plasma concentrations could be identified, nor with pathological treatment response.

**Abstract:**

Imatinib plasma trough concentrations are associated with efficacy for patients treated for advanced or metastatic KIT-positive gastrointestinal stromal tumours (GISTs). This relationship has not been studied for patients treated in the neoadjuvant setting, let alone its correlation with tumour drug concentrations. In this exploratory study we aimed to determine the correlation between plasma and tumour imatinib concentrations in the neoadjuvant setting, investigate tumour imatinib distribution patterns within GISTs, and analyse its correlation with pathological response. Imatinib concentrations were measured in both plasma and in three regions of the resected primary tumour: the core, middle part, and periphery. Twenty-four tumour samples derived from the primary tumours of eight patients were included in the analyses. Imatinib tumour concentrations were higher compared to plasma concentrations. No correlation was observed between plasma and tumour concentrations. Interpatient variability in tumour concentrations was high compared to interindividual variability in plasma concentrations. Although imatinib accumulates in tumour tissue, no distribution pattern of imatinib in tumour tissue could be identified. There was no correlation between imatinib concentrations in tumour tissue and pathological treatment response.

## 1. Introduction

Gastrointestinal stromal tumours (GISTs) are the most common, though rare in occurrence, mesenchymal tumours that arise throughout the gastrointestinal tract, predominantly in the stomach (50–60%) or small intestines (20–30%) [1]. The majority of GISTs harbour activating mutations in KIT (60–70%) or platelet-derived growth factor receptor α (PDGFRA) (10–15%) [2]. Imatinib is the standard treatment option for unresectable, locally advanced, and metastatic KIT-positive GISTs [3]. In the case of localized GISTs, complete surgical resection of the tumour remains the only potentially curative treatment. In locally advanced GIST neoadjuvant imatinib treatment for 6–12 months is recommended to reduce the tumour volume and, therefore, optimize the chance for successful curative surgery with less morbidity. Only for patients with a high risk of disease recurrence, adjuvant treatment with imatinib is indicated [3]. Imatinib is mainly metabolized by the cytochrome P450 (CYP) 3A4 and predominantly excreted via de biliary-faecal route [4].

For the treatment of advanced or metastatic GISTs, it has been demonstrated that imatinib trough plasma concentrations above 1100 μg/L at steady state conditions are associated with longer treatment benefit [5]. Nevertheless, there is a lack of understanding whether plasma concentrations also correlate with tumour drug concentrations. Assuming that there is a correlation, one might hypothesize that inadequate systemic exposure may lead to inadequate exposure at the target site, which can ultimately lead to acquired imatinib resistance. This hypothesis is currently under investigation as part of the GALLOP study, NCT02331914 [6]. One strategy to minimize treatment failure as a result of inadequate drug exposure is therapeutic drug monitoring (TDM)-guided dosing.

However, even in patients with plasma concentrations exceeding the threshold, resistance is still unavoidable [7,8]. It has been proposed that alterations in transporters, affecting tumour drug pharmacokinetics, might also play an important role in imatinib resistance [9,10]. This hypothesis is strengthened by the observation that intracellular imatinib concentrations are found to be lower in resistant GIST cell lines compared with parental cell lines [11]. More importantly, this finding was confirmed in three resistant GIST patients showing significantly lower tumour imatinib concentrations compared to three sensitive GIST patients [11]. For the measurement of tumour drug concentrations, tumour tissue has to be grinded or lysed whereafter the drug concentration can be measured in tissue homogenate. With the use of advanced analytical techniques, it becomes possible to investigate the spatial distribution of drugs in tissue [12,13]. However, it still remains impossible to determine the actual location as well as the concentration of imatinib in various cell compartments intracellularly.

We hypothesize that a wide range of factors may complicate imatinib tumour penetration, such as decreased tumour vascularity, treatment-induced necrosis, or differential expression of transporters [13,14]. To date, there is, however, a paucity of data regarding imatinib distribution in tumour tissue and whether or not tumour imatinib concentrations are correlated with plasma concentrations. The neoadjuvant setting is the ideal setting to explore this, as tumour resection is the standard treatment in patients with localised GISTs. Therefore, in this exploratory study we aimed to explore the correlation between plasma and tumour imatinib concentrations, to investigate tumour imatinib distribution within GISTs after neoadjuvant treatment with imatinib and its correlation with pathological response to treatment.

## 2. Materials and Methods

### 2.1. Study Design and Population

**Figure 1:**
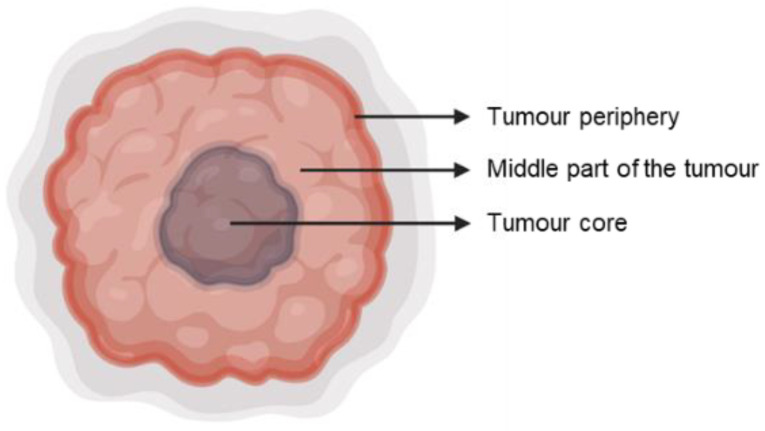
Schematic overview of different tumour regions.

In this exploratory study, patients treated with imatinib for GIST in the neoadjuvant setting and candidates for tumour resection according to standard care were asked to participate. Patient and tumour characteristics were collected from the electronic patient records, as well as the imatinib starting dose, the imatinib dose prior to surgery, treatment duration, mutational status, and pathological treatment response defined as the percentage of tumour necrosis or hyalinization as determined by a sarcoma pathologist [15]. In all patients, one blood sample was collected directly before surgery to quantify imatinib plasma trough concentrations. Directly after tumour resection, a total of three samples from three different tumour regions were retrieved by a pathologist. These included tissue from the (frequently more necrotic) core, the middle part, and the (more vital) tumour periphery (Figure 1). Tissue samples were weighed directly after sample collection whereafter both tissue and blood samples were stored at <−20 °C until analyses. All patients provided written informed consent.

### 2.2. Sample Preparation and Quantification

The tumour tissue samples were weighed and homogenised with beads in 1 mL of DMSO. After homogenization, both an undiluted and 10-times-diluted sample were prepared. For the quantification of imatinib in plasma and tumour tissue, a validated liquid chromatography-tandem mass spectrometry (LC-MS/MS) assay was used in a similar manner to a previously described method, with minor modifications [16]. Detailed information regarding the analysis is described in the Supplementary File.

### 2.3. Data Analysis

For each plasma sample, the date and time of the last imatinib intake and date and time of blood sampling were recorded. The plasma trough concentration was estimated using log-linear extrapolation based on the elimination half-life of imatinib and time after dose, as previously described [17]. In order to investigate a possible correlation between plasma and tumour imatinib concentrations, a plasma density factor was used to convert plasma concentrations into ng imatinib per mg plasma. The density factor was calculated by weighing 1 mL of plasma seven times. Subsequently, the average density in g/L was used for further calculations. The average tumour concentration of the three tumour regions was calculated for each tumour sample. Spearman’s rho was used to test for a possible correlation between average tumour tissue and plasma concentration, as well as the average tumour tissue concentration and treatment response. The latter was scored as suggested by the European Organization for Research and Treatment of Cancer Soft Tissue and Bone Sarcoma Group (EORTC-STBSG) [15]. A *p*-value < 0.05 was considered to be statistically significant.

Imatinib tumour concentrations in different tumour regions within the same tumour sample were evaluated to investigate the distribution pattern of imatinib within GISTs. Results below the lower limit of quantification (<LLOQ) were replaced by the value LLOQ/2 making it possible to handle these data in the analysis, which can be considered a common approach in clinical pharmacology studies [18,19]. Both intertumoral variability on the average tumour concentration per patient and interpatient variability in plasma concentration were calculated and described. To explore whether the average imatinib tumour tissue concentrations were statistically different compared to imatinib plasma concentrations, a paired *t*-test was performed. A tumour-to-plasma ratio was calculated for each patient using the average imatinib tumour tissue concentrations and the plasma trough concentration.

## 3. Results

Between August 2019 and December 2022, eight patients with GIST who were treated with neoadjuvant imatinib, and for whom tumour resection was indicated, were included in this study. Patient and tumour characteristics are shown in Table 1. Patients were treated with 400–600 mg imatinib OD until the day before surgery. Primary tumours originated from either the stomach (n = 5), cardia (n = 2), or rectum (n = 1). Six patients were treated with imatinib in the neoadjuvant setting for a localized GIST. Two patients had liver metastases that were resected as well. The median time on imatinib treatment before surgery was 11 months (IQR 8–12).

Some patients used comedications for the treatment of comorbidities as listed in Table 1, but none were interacting with imatinib. Patient 7 developed a moderate hepatic impairment due to liver metastases (bilirubin > 1.5–3.0× upper limit of normal and elevated transaminases). Both bilirubin and transaminases normalized after tumour resection.

**Figure 2:**
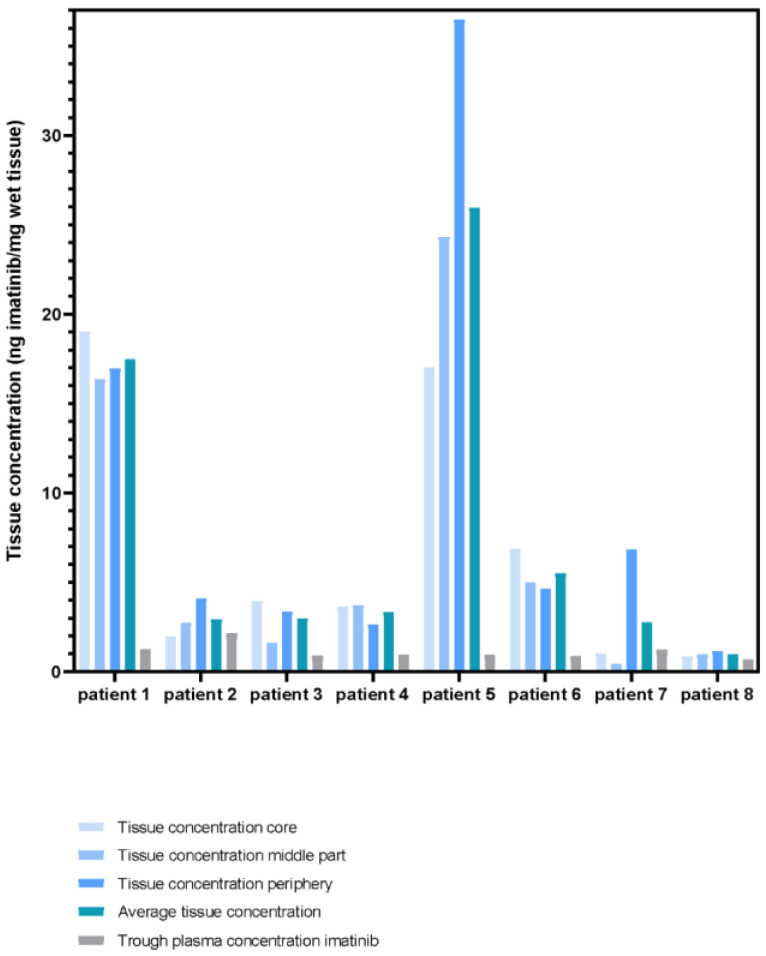
Imatinib concentrations in primary tumour tissue lesions and plasma.

**Figure 3:**
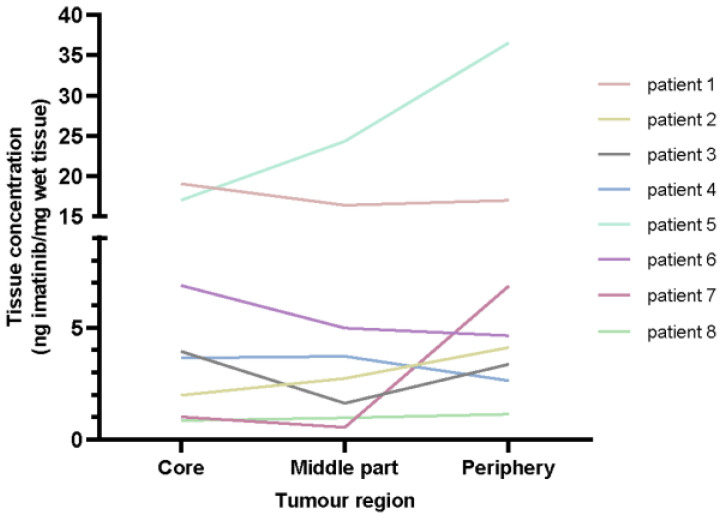
Imatinib concentrations in different primary tumour tissue regions.

An overview of measured tumour imatinib concentrations as well as the plasma concentrations are presented in Figure 2 and described in Supplementary Table S1. In four out of twenty-four primary tumour samples, the quantified imatinib concentration was below the lower limit of quantification (LLOQ; <50 ng/mL). To be able to incorporate these samples within the analysis, the values were replaced with 25 ng/mL (LLOQ/2). The median of all primary tumour samples, derived from eight tumour lesions, was 4.0 (range 1.0–36.5) ng imatinib per mg wet tissue. Figure 3 shows the distribution pattern of imatinib in the different tumour regions for the individual patients. No general distribution pattern of imatinib in GISTs could be identified. In the primary tumours of patients 2, 5, and 7, imatinib concentrations in the tumour periphery were more than two times higher compared to the concentration in the tumour core. Although less pronounced, for patient 4 and 6 the imatinib concentration in the tumour core appeared to be higher than in the tumour periphery. In the other patients no remarkable differences could be identified, suggesting an equal distribution of imatinib across the different tumour regions.

**Figure 4:**
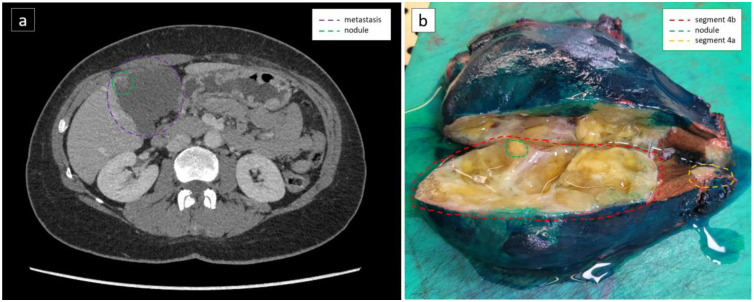
Liver metastasis on CT scan and after resection: (**a**) CT scan two months prior to surgery. The purple encircled part represents the complete liver metastasis. The green encircled part represents the regenerative nodule in a mass; (**b**) Cut-open resection of liver segments 4a and 4b, encircled in yellow and red, respectively. Segment 4b contains the regenerative liver ‘nodule within the mass’, encircled in green, directly after surgery.

In two patients, imatinib concentrations were measured in both primary tumour and liver metastases. In patient 7 the average tumour concentrations in the primary tumour and the liver metastases were 2.8 and 6.8 ng imatinib per mg wet tissue, respectively. For patient 8, the average tumour concentrations in the primary tumour and the two liver metastases, liver segment 4a and 4b, were 1.0, 2.1, and 1.4 ng imatinib per mg wet tissue, respectively. Based on a CT scan two months prior to surgery, there appeared to be a recurrent vital nodule (‘nodule within the mass’) present within the metastasis located in liver segment 4b (Figure 4). Therefore, the pathologist attempted to sample this nodule. An imatinib concentration of 1.7 ng imatinib per mg wet tissue was measured in this nodule.

**Figure 5:**
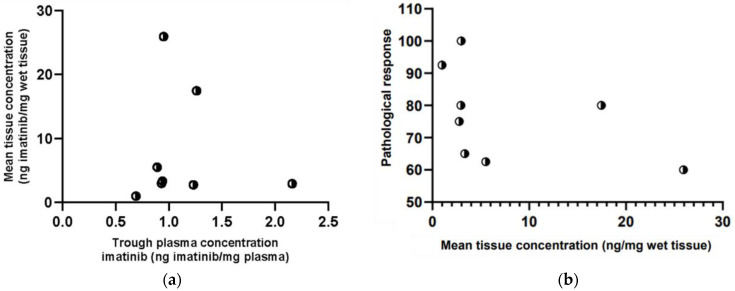
(**a**) Correlation between imatinib plasma and average primary tumour concentrations; (**b**) Correlation between imatinib average primary tumour concentrations and pathological treatment response.

The measured plasma concentration prior to surgery ranged from 700 to 2175 μg/L (median 954 μg/L). The calculated plasma density factor was 1009 g/L, resulting in a median plasma concentration of 0.9 (range 0.7–2.2) ng imatinib per mg plasma. A mean (±sd) tumour-to-plasma ratio of 7.4 ± 9.0 was found. There was no statistically significant difference between mean tumour imatinib concentrations and imatinib plasma concentrations (*p* = 0.077). No correlation was found between imatinib tumour tissue concentrations and plasma concentrations (ρ = 0.190, *p* = 0.651) (Figure 5a), nor with pathological treatment response (ρ = −0.599, *p* = 0.117) (Figure 5b). Intertumoural variability on the average tumour concentration per patient (coefficient of variation (CV) 116%) was almost three times greater compared to interpatient variability in plasma concentration (CV 42%).

## 4. Discussion

In this exploratory study, mean tumour imatinib concentrations were found to be higher compared to imatinib plasma concentrations. No correlation was found between plasma and tissue imatinib concentrations. Despite relatively low interpatient variability in plasma concentrations, high differences were observed in tumour imatinib concentrations between patients. No general distribution pattern of imatinib within primary GISTs could be identified. No correlation was found between imatinib tissue concentrations and pathological treatment response.

The median plasma concentration of 954 μg/L prior to surgery seems to be comparable with plasma concentrations described in other real-life cohorts. In both TDM and non-TDM cohorts, median plasma concentrations of 756 to 1082 μg/L were reported [20,21,22,23,24]. The interpatient variability in imatinib plasma concentrations found in this study (CV 42%) is comparable to earlier findings reporting CVs from 38 to 75 % [22,23,25]. Furthermore, the median tumour imatinib concentration of 4.0 (range 1.0–36.5) ng imatinib per mg wet tissue is lower compared to earlier findings from Zhang et al. [11]. This study investigated imatinib tumour concentrations in six advanced GIST patients of whom half of the patients were imatinib sensitive and the other half imatinib resistant. The average imatinib tissue concentration was 15 and 10 ng imatinib per mg wet tissue for the responders and non-responders, respectively [11]. In a study of Berglund et al., imatinib concentrations were measured in three patients. In two patients different tumour regions within the same tumour were measured and it was found that imatinib concentrations increased from the tumour core towards the periphery in both patients. Tumour imatinib concentrations measured in our study were comparable to imatinib concentrations measured in this study, ranging from 0.4 to 36.6 ng imatinib/mg wet tissue [26]. Although the highest imatinib concentration in our study was found in the tumour periphery as well, we clearly showed that the distribution pattern differs between patients. In our data it was not possible to identify a general distribution pattern of imatinib within the tumour. 

In all tumour lesions the mean imatinib concentration outweighed the corresponding plasma concentration with an overall mean tumour-to-plasma ratio of 7.4. Theou et al. found similar results in experiments with mice, as imatinib tissue concentrations were 6- to 8-fold higher compared to plasma [27]. Berglund et al. investigated both plasma and tissue imatinib concentrations in three patients with advanced GIST who were treated with imatinib 400 mg once daily in the neoadjuvant setting [26]. In line with our data, the authors report tissue concentrations ~5.3- to 9.6-fold higher compared to plasma. Our data together with previous findings demonstrate higher imatinib concentrations in tumours compared to plasma, indicating an accumulation of imatinib in tumour tissue. The accumulation of TKIs in tumour tissue has been described before with tumour-to-plasma ratios ranging from 0.6 to 178 [28,29,30,31,32,33]. Although accumulation in tumour tissue is highly drug-dependent and possibly also tumour-type-dependent, it is unknown which factors are responsible for this finding. Drug characteristics such as the molecular mass, acid dissociation constant pKa, and partition coefficient logP might play an important role. In addition, the drug’s affinity for drug transporters that are expressed at tumour cells may be important, and some drugs can even undergo lysosomal sequestration, as is the case for sunitinib [34]. Although it can be speculated that high TKI concentrations at the tumour are beneficial, it should be kept in mind that intracellular concentrations measured using tissue homogenates do not reflect the effective drug concentration at its target site. For drugs that accumulate in cells, measurement of total tissue levels may lead to underestimation of the actual intracellular concentration since the analysis is performed on a mixture of both intra- and extracellular fluids.

Surprisingly, no correlation was found between plasma and tumour concentrations, indicating that imatinib does not freely diffuse within the tumour and that other factors may play a role in the inequal distribution of imatinib over tumour tissue. We speculate that other factors, such as lysosomal sequestration and a reduction in tumour vascularization, may play a dominant role in the distribution of imatinib. Abu Sammour et al. confirmed the latter hypothesis with their findings, showing a decreased vasculature in GIST liver metastasis, mostly in the tumour core, compared to normal liver tissue after imatinib treatment [13]. They showed that almost no imatinib appeared to be detectable in this liver metastasis, despite high imatinib concentrations in the normal surrounding tissue. No plasma concentrations were measured in their study. Although in our study imatinib tumour concentrations were found to be higher in liver metastases compared to the corresponding primary tumours, no concentrations were measured in surrounding healthy tissue making it impossible to compare these results. In general, it could be hypothesized that, due to a reduction in tumour vascularization imatinib penetration in tumour tissue could be hampered, potentially leading to ineffective treatment. In addition, although mostly investigated in chronic myeloid leukaemia (CML) cells, imatinib is reported to be prone for lysosomal sequestration due to its physiochemical properties [35,36,37]. This finding was confirmed by a study by Burger et al., who report an accumulation of imatinib in acidic lysosomal compartments in GIST cell lines as well [38]. In these cell lines, imatinib remained to effectively inhibit c-KIT. It has been described that the cellular efflux of imatinib is mediated by p-glycoprotein (ABCB1), more frequently expressed on gastric tumours compared to non-gastric tumours [39,40]. There is, however, an ongoing debate about whether or not ABC transporters are also expressed on lysosomal membranes intracellularly [36,41]. If so, it could be hypothesized that this would facilitate lysosomal trapping of imatinib, eventually resulting in high tumour concentrations after mechanical disruption of the tissue.

Although, to date, no standardized pathologic evaluation criteria of GISTs exist, the EORTC-STBSG has proposed a soft-tissue sarcoma response score based on the proportion of vital tumour cells [15]. With the use of this system, the percentage of treatment response in the primary GISTs was scored by the pathologist. Thereby, an association between tumour imatinib concentrations and pathological treatment response was made. However, the exact percentage of response per biopsied tumour region remains unknown, which may have complicated investigating this correlation. Furthermore, it is impossible to differentiate imatinib-induced tumour regression from primary regressive changes prior to the start of imatinib treatment, potentially explaining the lack of an association. 

A limitation of our study is that it remains unknown where exactly in the tissue imatinib is located. The measurement of imatinib concentrations in homogenized tumour tissue is a sum of intra- and extracellular fluids. Imatinib might be unequally distributed in different cell compartments. It is unknown to which extent imatinib reached the target site intracellularly. Additionally, due to the heterogeneity of the tumours with regards to its size and shape, it is difficult to distinguish the different regions of the tumour. This is especially the case for small tumour samples. However, biopsies from tumour resections were taken by trained pathology analysts in a sarcoma centre, thereby also reducing sample errors. Nevertheless, the ability to sample three different regions of the tumour due to complete resection is also a strength of this study, as we were, thereby, able to explore imatinib tumour distribution. It is important to highlight that in the present study all patients were treated in the neoadjuvant setting. It is unknown if imatinib keeps accumulating in tumour tissue after prolonged treatment or if a plateau phase is reached. Since we only measured tissue concentrations at one time point it remains unknown if the imatinib tumour concentrations in the adjuvant and metastatic setting will be in the same range as in the neoadjuvant setting. Although the sample size of this study is relatively small, the sample size is deemed sufficient to conclude that a distribution pattern of imatinib in GISTs cannot be identified.

## 5. Conclusions

This exploratory study revealed that imatinib concentrations in tumour tissue outweighed plasma concentrations and that no correlation was observed between plasma- and tumour concentrations. Interpatient variability in tumour concentrations was high compared to interindividual variability in plasma concentrations. Although imatinib accumulates in tumour tissue, no distribution pattern of imatinib in tumour tissue could be identified. We hypothesize that other factors such as lysosomal sequestration, tumour necrosis, and a reduction in tumour vascularization may be important factors involved in tumour distribution of imatinib. In the literature, imatinib plasma concentrations seem to be highly correlated with efficacy. However, the results of this study highlight that there is no correlation between imatinib tumour tissue concentrations and pathological treatment response. Based on the results of this study, measuring tumour imatinib concentrations in GISTs seems to be redundant.

## Figures and Tables

**Table 1:** Patient and tumour characteristics.

	Patient 1	Patient 2	Patient 3	Patient 4	Patient 5	Patient 6	Patient 7		Patient 8		
Patient Characteristics											
**Gender**	Male	Male	Male	Female	Male	Male	Female		Female		
**Age at time of surgery (*years*)**	50	63	55	71	72	61	60		52		
**Length at time of surgery (*cm*)**	177	183	185	163	176	165	167		163		
**Weight at time of surgery (*kg*)**	79	127	93	106	84	110	66		68		
**ECOG performance status**	0	0	1	0	1	0	0		0		
**Metastasis**	No	No	No	No	No	No	Yes		Yes		
**Tumour site**	Stomach	Rectum	Stomach	Cardia	Stomach	Cardia	Stomach	Liver metastasis	Stomach	Liver metastasis segment 4a	Liver metastasis segment 4b
**Days from diagnosis untill start imatinib**	15	62	10	7	15	20	12		24		
**Treatment duration (*months*)**	12	8	8	11	11	9	34		19		
**Imatinib starting dose (*mg/day*)**	400	400	400	400	400	400	400		400		
**Imatinib dose prior to surgery (*mg/day*)**	600	400	500	400	400	600	600		400		
**C_trough_ level imatinib prior to surgery (*μg/L*)**	1275	2175	935	950	957	897	1238		700		
**eGFR at start imatinib (*ml/min/1.73 m^2^*)**	>90	72	85	65	66	>90	>90		77		
**eGFR at time of surgery (*ml/min/1.73 m^2^*)**	>90	55	74	58	71	70	>90		79		
**Hepatic function at start imatinib ^**	normal	normal	normal	normal	normal	normal	normal		normal		
**Hepatic function at time of surgery ^**	normal	normal	normal	normal	normal	normal	moderate		normal		
**Albumin levels at start imatinib (*g/L*)**	40	35	36	36	38	39	35		37		
**Albumin levels at time of surgery (*g/L*)**	40	35	37	38	34	37	24		37		
**Comorbidities**	none	Atrial fibrillation, hypercholesterolaemia, hypertension	Cerebrovascular accident	Type 2 diabetes, hypertension	Prostate carcinoma, hypercholesterolaemia, hypertension	Hypertension, triple vessel disease	none		none		
**Tumour characteristics**											
**Tumour diameter at time of diagnosis (*cm*)**	4.0	6.0	9.3	12.0	7.2	16.6	17.2	8.5	4.5	unknown	12.2
**Tumour diameter at time of surgery (*cm*)**	3.0	5.0	8.5	7.7	5.5	10.5	11.0	4.5	2.0	unknown	0.9
**Mutational status (*cKIT*)**	exon 11	exon 11	exon 11	exon 11	exon 11	exon 11	exon 11		exon 11		
Treatment response * (*%*)	80	80	100	60–70	60	50–75	70–80	unknown	90–95	100	90

^ As categorized according to the National Cancer Institute Common Toxicity Criteria for Adverse Events, version 4.0 (CTCAEv4) and Organ Dysfunction Working Group: normal, mild, moderate, or severe. * Scored by a pathologist in percentages, based on the degree of tumour necrosis or hyalinization as proposed by the EORTC-STBSG.

## Data Availability

The authors confirm that the data supporting the findings of this study are available within the article and its Supplementary Materials.

## Supplementary Materials

The following supporting information can be downloaded at https://www.mdpi.com/article/10.3390/cancers15112875/s1. File S1: quantification of imatinib in tumour tissue; Table S1: imatinib concentrations in the different tumour regions of the primary tumour, metastases, and plasma.
