# Supplementary material for: Measuring Tumour Imatinib Concentrations in Gastrointestinal Stromal Tumours: Relevant or Redundant?
Source: Cancers (Basel). 2023 May 23;15(11):2875. doi: 10.3390/cancers15112875 (PMC10251911; doi:10.3390/cancers15112875)
Supplement: Supplementary file 1 [file cancers-15-02875-s001.zip › Supplementary File S1.pdf]

Supplementary File S1

Detection of imatinib took place at the mass transitions 494.3 > 394.2 (precursor > collision ion product). Each analytical run included a calibration curve of imatinib spiked in plasma over the concentration range of 50–50,000 μg/L.

Quality controls (QCs) were analysed in duplicate at three different concentrations of 150, 2000 and 37,500 μg/L. The stable isotope imatinib [13C,2H3] was used as internal standard (IS).

Each tissue sample of 15-50 milligrams was weighed and thereafter transferred into a Precellys vial with beads (Precellys lysing Kit Hard tissue homogenizing CK28-R®, Bertin Technologies) and 1.0 millilitre (mL) of DMSO. After homogenization, both an undiluted and 10 times diluted sample were prepared. For the undiluted sample, 50 microliters (μL) of the homogenate was transferred into a 1.5mL vial together with 200μL of precipitation reagent (methanol) and internal standard. To make the 10 times diluted sample, 5μL of homogenate was transferred into a 1.5mL vial together with 45μL of blank plasma and 200μL of precipitation reagent (methanol). For the preparation of plasma samples, 50μL of plasma was transferred into 1.5mL vials together with 200μL of precipitation agent (methanol) and internal standard. The samples were centrifuged for 5 minutes at 18,600 g. Imatinib was quantified in 100 μL supernatant.

Concentrations of imatinib were determined using a validated ultra-performance liquid chromatography with mass spectrometry detection (LC-MS/MS) assay similar to a previously described method, with minor modifications [1]. The column used was an Acquity UPLC® BEH C18 1,7 μm 2,1 x 100 mm (Waters®, Milford, USA) maintained at 50°C. A combination of phase A (2 mM acetate water buffer) and phase B (2 mM acetate methanol buffer) was used as mobile phase which was delivered in a gradient. Phase B was initiated at 25% and held for 1 min, then increased linearly to 50% at 3 min, 90% at 5 min, and held at 90% at 8 min, then back to 25% until the end of the run of 9 min at a flow rate of 0.4 ml/min. The needle was washed with 1% (v/v) acetonitrile for 5 sec after each injection.

A minimum of two sets of QCs were measured in each LC-MS/MS run. The results of the dilution integrity showed that the accuracy and precision for 10 times diluted test samples were within the acceptance criteria of ±15%. All samples were measured in duplicate. Tumour imatinib concentrations were determined by back calculation to the calibration curve. With the use of the sample weight the result could be expressed as ng imatinib per mg wet tissue.

References

1. van Erp NP, de Wit D, Guchelaar HJ, Gelderblom H, Hessing TJ, Hartigh J. A validated assay for the simultaneous quantification of six tyrosine kinase inhibitors and two active metabolites in human serum using liquid chromatography coupled with tandem mass spectrometry. J Chromatogr B Analyt Technol Biomed Life Sci. 2013;937:33-43.
