# Supplementary material for: Measuring Tumour Imatinib Concentrations in Gastrointestinal Stromal Tumours: Relevant or Redundant?
Source: Cancers (Basel). 2023 May 23;15(11):2875. doi: 10.3390/cancers15112875 (PMC10251911; doi:10.3390/cancers15112875)
Supplement: Supplementary file 1 [file cancers-15-02875-s001.zip › Supplementary Table S1.pdf]

Supplementary Table S1

**Patient**	**Tumour tissue concentration imatinib (ng/mg wet tissue)**					**Trough plasma concentration imatinib (ng/mg plasma)**
	Primary tumour				Metastasis	
	Core	*Middle part*	*Periphery*	*Mean*	*Mean*	
1	19.0	16.4	17.0	17.5	-	1.3
2	2.0	2.7	4.1	2.9	-	2.2
3	3.9	1.6	3.4	3.0	-	0.9
4	3.6	3.7	2.6	3.3	-	0.9
5	17.0	24.3	36.5	25.9	-	0.9
6	6.9	5.0	4.6	5.5	-	0.9
7						
primary	1.0	0.4	6.9	2.8	-	1.2
metastasis	-	-	-	-	6.8	
8						
primary	0.9	1.0	1.1	1.0	-	0.7
metastasis 4a	-	-	-	-	2.1	
metastasis 4b	-	-	-	-	1.4	
nodule	-	-	-	-	1.7	

<LLOQ below the lowest limit of quantification
